# Chiari malformation type 1 is associated with a smaller fourth ventricle volume – a multi-cohort replication study

**DOI:** 10.1038/s41598-026-56778-x

**Published:** 2026-06-21

**Authors:** Jarod L Roland, Thanda Meehan, Alexandra Gabliani, Scott Marek, Nico Dosenbach, Joshua S Shimony, Nilay Jakati, Yi Li, Leo Sugrue, Jennifer M Strahle, Chenyang Lu, David D Limbrick, Gabe Haller

**Affiliations:** 1 Taylor Family Department of Neurosurgery, Washington University School of Medicine, St. Louis, MO USA; 2 AI for Health Institute, Washington University in St Louis, St. Louis, MO USA; 3 Department of Radiology, Washington University School of Medicine, St. Louis, MO USA; 4 Department of Neurology, Washington University School of Medicine, St. Louis, MO USA; 5 Department of Radiology, University of California San Francisco, San Francisco, CA USA; 6 Department of Computer Science and Engineering, Washington University School of Medicine, St. Louis, MO USA; 7 Department of Neurological Surgery, Virginia Commonwealth University, Richmond, VA USA

**Keywords:** Chiari I malformation, Ventricle, Magnetic resonance imaging, Segmentation, Anatomy, Medical research, Neurology, Neuroscience

## Abstract

Chiari Malformation Type 1 (CM1) is canonically defined by ectopic position of the cerebellar tonsils with additional anatomic variations described inconsistently. Effect on fourth ventricle volume is controversial with prior studies reporting disparate results and methodologically limited to single institution series that hinder generalizability. This limitation was addressed utilizing multiple data sources, longitudinal replication, and reproducible methods using both FreeSurfer and the deep learning-based DL+DiReCT tools for automated volumetrics. First, we analyzed a local retrospective clinical cohort of individuals with CM1 and controls. Using data from the Adolescent Brain Cognitive Development (ABCD) Study, we analyzed volumes at baseline then replicated at two longitudinal timepoints. We then utilized an independent deep learning tool to demonstrate reproducibility with ABCD baseline data. Next, we again replicated with a prospective cohort from the Redefining Chiari (RC) study and compared to controls from the Human Connectome Project Young Adult (HCP-YA) study. Finally, we analyzed a heterogenous dataset from the Park-Reeves Syringomyelia Research Consortium (PRSRC) with comparison to ABCD baseline controls. Our aim was to test the hypothesis of a relationship between fourth ventricle size and CM1. Across all datasets, timepoints, and segmentation tools, we consistently found CM1 associated with smaller fourth ventricle volume. Our findings robustly demonstrate that a smaller fourth ventricle volume is an anatomical feature associated with CM1 at the group level. Fourth ventricle volume in CM1 may provide additional insights into pathophysiology but will require further study to fully elucidate its clinical importance.

## Introduction

Contemporary understanding of the salient features of Chiari Malformation Type 1 (CM1) has been progressively refined since the initial classification was published by the Austrian pathologist Hans Chiari in 1891^1^. After this seminal introduction to tonsillar ectopia as a pathologic finding, additional descriptions of atypical hindbrain anatomy further expanded on the eponymous entity with sub-types of Chiari Malformation, initially described as types 1 through 4. Even more analysis continued to expand the numeric categorization with type 0, 1.5, and others^2,3^. In CM1 multiple other anatomic variations of the posterior fossa and craniocervical junction have been described in association with classical ectopic tonsil position and variably correlated with the wide range of symptoms that have been attributed to CM1^4^.

Cerebellar tonsil ectopia can be acquired secondary to other pathologies including hydrocephalus, posterior fossa tumor with direct mass effect on the adjacent cerebellum, and spinal CSF leak causing a negative pressure gradient leading to download displacement of posterior fossa contents. However, CM1 refers to cerebellar tonsil ectopia that is not secondary to another process such as a tumor or other mass occupying lesion. Therefore, the pathogenesis of this anatomic abnormality and how it may be responsible for the associated clinical phenotypes is a topic of ongoing clinical investigation.

There are multiple hypotheses addressing the relationship of CSF flow and tonsillar ectopia. These span a wide range including obstruction of fourth ventricle outflow through the foramen of Magendie^5–9^, volumetric mismatch between the posterior fossa and its contents (i.e., cerebellum, brainstem, and fourth ventricle)^10–13^, atlantoaxial instability^14,15^, abnormal cervical dura compliance^16^ and others. A scoping review of pathophysiology of Chiari Malformation and its various forms is beyond the scope of this report. While an assessment of the fourth ventricle volume alone is unlikely to fully resolve the quintessential debate between competing hypotheses on CM1 pathogenesis, its relationship may be one piece to a much larger puzzle. Unfortunately, scientific attempts thus far to firmly establish such a relationship have been inconsistent. Prior reports on fourth ventricle size in CM1 have suggested larger size^17,18^, smaller size^19^, and no difference^20^. We hypothesize that limitations in sample size and methodological heterogeneity, which are common confounds and well-described limitations of retrospective analysis^21^, may limit the replicability of previously published data. In this study we aim to overcome these limitations via strategic methodological techniques with big data analysis to reproduce and replicate our findings across multiple datasets and with independent automated volumetrics tools. Specifically, we investigate whether fourth ventricle volume is associated with CM1 by testing for difference relative to a control group using multiple data sources and automated analysis techniques.

## Methods

**Fig. 1:**
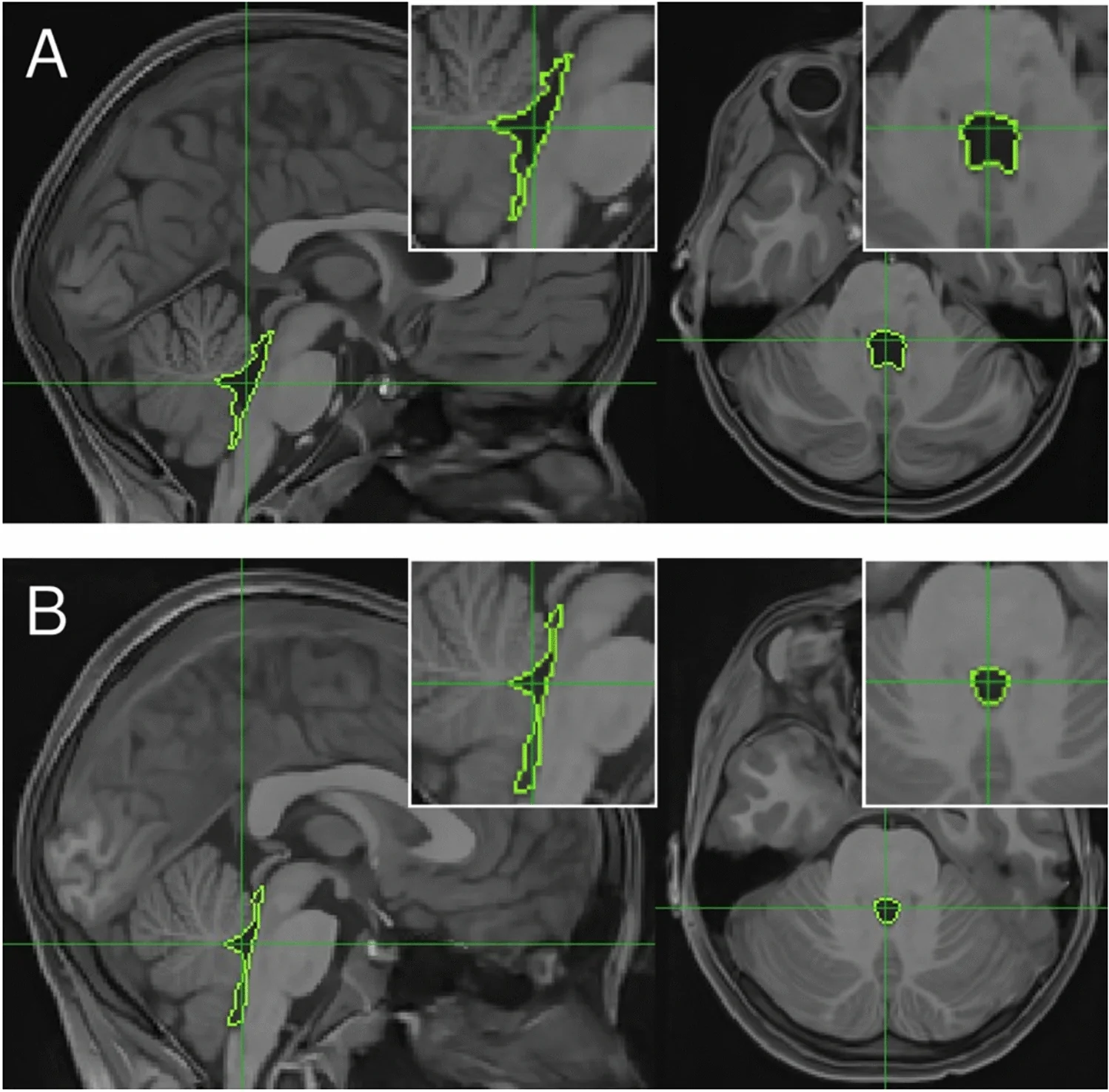
Fourth ventricle volume segmentation for exemplar individuals in the control (**A**) and CM1 (**B**) groups. Mid-sagittal images on left and axial through the widest anterior-posterior dimension of the fourth ventricle on the right. Inset magnified area of fourth ventricle.

We hypothesized that individuals with CM1 have smaller fourth ventricle volumes than controls. To test this hypothesis, we collected MRI data from multiple independent sources for replication and reproduction. This study received ethical research approval by the Institutional Review Board (IRB) at Washington University in St Louis (WashU) and all experiments were performed in accordance with relevant guidelines and regulations. Prospectively enrolled individuals of the Redefining Chiari Study provided informed consent. Retrospective clinical data from the SLCH cohort and the Park-Reeves Chiari and Syringomyelia Research Consortium (PRSRC) was obtained via waiver of consent as approved by the relevant guidelines and regulations of the WashU IRB. Participants from the ABCD Study and HCP-YA Study were consented per their study protocol and shared via an open science method. Imaging studies from the ABCD Study, HCP, and RC Study underwent prior quality screening at acquisition and did not require further screening for this study. Images from the SLCH local cohort were selected to be age and sex matched with high resolution MRI data and therefore did not require further screening. Images from the PRSRC were collected from the database first by searching for high resolution brain MRIs. The 184 initial results were then manually screened and 91 were rejected for either poor quality, evidence or prior surgery including posterior fossa decompression, ventricular shunt, other cranial surgery, or other mass occupying lesion. The processing parameters, registration pipelines, and runtime platforms have been previously described for the ABCD Study^22,23^ and HCP-YA^24^ datasets. The software pipeline for the Redefining Chiari Study mimics that of the ABCD by using the official ABCD-HCP-pipeline v0.1.4 of the Docker container. We used two independent software tools (FreeSurfer and DL+DiReCT) that perform automated brain segmentation to define a 3D volume of the fourth ventricle for group level analysis (Fig. 1). Subcortical segmentation by FreeSurfer is performed as part of the larger recon-all pipeline, which has been developed and refined over many iterations. The process involved aligning the subject’s T1 weighted image to a standard space where a probabilistic atlas of subcortical structures can be utilized. Bayesian inference is performed to assign label probabilities to voxels based on voxel intensity values and spatial priors. While there are many layers to FreeSurfer segmentation and parcellation, the initial subcortical segmentation was described by Fischl and colleagues in 2002^25^. Importantly, they describe a method that is robust to variation of pathologic anatomy, including morphological differences specifically tested in the setting of Alzheimer’s disease. When assessing differences between cohorts only volumes computed by the same method were compared (i.e., FreeSurfer to FreeSurfer or DL+DiReCT to DL+DiReCT, but never FreeSurfer to DL+DiReCT). We searched the electronic medical records at St Louis Children’s Hospital (SLCH) to identify individuals with a clinical diagnosis of CM1 and high-quality MRI data. We screened for T1 weighted images with at least 1 mm isotropic voxel resolution. We manually evaluated each image to exclude those with evidence of prior surgery, shunts or other implants, unrelated anatomic abnormalities such as arachnoid cyst, or other mass occupying lesions for exclusion. We similarly screened records for a comparable group of clinical imaging with the same criteria for use as a control group.

From the Adolescent Brain Cognitive Development (ABCD) Study we collected tabulated data from the National Data Archive (NDA) data release version 6.0. A subset of the T1-weighted MRI volumes was downloaded and processed for replication of the tabulated NDA results. The incidental CM1 subjects were identified based on the safety reads performed by trained pediatric neuroradiologists as part of the study’s data collection protocol and reported previously along with other incidental findings in the ABCD Study^26^. Previously published data on inter-rater reliability in CM1 has shown a non-trivial range when measuring tonsillar ectopia^27,28^, implying variability in diagnosis. We acknowledge that other studies have reported a different number of individuals with CM1 in the ABCD Study^29^. However, we are unable to replicate those methods, and their list of cases is not published, therefore we focused our analysis on the individuals identified by two board certified neuroradiologists during safety reads for the ABCD Study. For the control cohort in our replication analysis, we downloaded a subset of ABCD data that was described as a discovery dataset in prior studies by selecting for known good quality imaging^30^. At the time of this study, complete data for the baseline timepoint was available and partial imaging data for the years 2 and 4 longitudinal timepoints was available.

The Redefining Chiari (RC) Study prospectively enrolled pediatric and adult participants with a clinical diagnosis of CM1 for high quality standardized MRI acquisition. T1-weighted images were acquired on a Siemens Prisma scanner, which is the same make and model as the most common of three scanner types used in the ABCD Study. To maximize similarity the MRI sequence and parameters used in the RC Study are identical to those of the ABCD Study^22,23^.

The Park-Reeves Chiari and Syringomyelia Research Consortium (PRSRC) is a multi-site research consortium that collects retrospective clinical imaging from individuals with CM1 and syringomyelia. From the PRSRC we screened for high-quality MRI data in a similar manner as our single site cohort, but with the added heterogeneity that is to be expected from multiple sites with myriad clinical imaging protocols. We first screened for resolution of at least 1 mm isotropic voxels then manually assessed for signs of prior surgery, shunted hydrocephalus, or unrelated anatomic pathology for exclusion.

The Human Connectome Project Young Adult (HCP-YA) study has made openly available MRI imaging data from individuals aged 22–25 years^31^. Volumetric measurements of the fourth ventricle from 1,113 individuals in the HCP-YA cohort were used as controls for comparison with the RC Study in this analysis.

The ABCD Study makes available a large corpus of pre-processed data via the National Data Archive (NDA) for download by investigators in tabulated format. Data from outputs of the FreeSurfer *recon-all* pipeline are available in machine parsable plain text format. FreeSurfer is free open-source software (FOSS) that performs multiple different functions, of which one is automated measurements of volumes for multiple brain structures. The ABCD Study NDA data includes volumetric outputs generated from FreeSurfer version 5.3^23^. We used the ABCD HCP-derived pipeline for pre-processing data from the RC Study to obtain similar volumetric measurements using the same version of FreeSurfer as the ABCD Study NDA data^32^.

Head size is previously reported confound for cerebral volumetric correlations^1^. For this reason, we corrected the fourth ventricle volume by the division method using the estimated total intracranial volume (eTIV) as reported by FreeSurfer. We also analyzed the raw fourth ventricle volume measurements for ease of interpretability of the volume as opposed to a unitless corrected ratio. We then used DL+DiReCT, a previously trained deep learning algorithm available as FOSS that produces volumetric segmentation outputs comparable to FreeSurfer but from an independent processing pipeline^33^. This served the purpose of demonstrating non-dependence on a specific volumetric segmentation technique, as well as benefiting from quicker processing gained from speed increases inherent to the deep learning approach. However, this pipeline does not produce an estimated total intracranial volume and therefore the corrected fourth ventricle volume ratios where all computed from FreeSurfer output.

Statistical analysis and data visualization was performed with Python version 3.11.9 and the software libraries SciPy^34^, NumPy^35^, Pandas^36^, psymatch^37^, and Seaborn^38^. Scripts to reproduce this analysis are available on GitHub at https://github.com/jarodroland/FourthVentricleVolumeCM1. The DL+DiReCT pipeline^33^ is shared publicly at https://github.com/SCAN-NRAD/DL-DiReCT. All computational tools used in this analysis are FOSS, thereby maximizing the ability for other researchers to replicate our results with their own data. Figures are plotted with a strip plot to display every data point, a violin plot visualizing the distribution, and a box plot with the 50^th^ percentile (median) marked by white line, 25^th^ and 75^th^ percentiles marked by the ends of the box, and whiskers extending to 1.5 times the inter-quartile range. Student’s T-test was used for inferential statistics on normally distributed data with a p-value of 0.05 as an a-priori threshold of statistical significance. To correct for multiple comparisons, we used the Benjamani-Hochberg procedure for controlling the false discovery rate. Effect size was measured by Cohen’s d for independent samples.

## Results

High quality MRI scans were collected, and volumetric segmentations were performed, for 68 individuals from the SLCH cohort (34 CM1 and 34 Controls), 11,755 individuals from the ABCD Study (71 CM1 and 11,684 Controls), 55 individuals from the Redefining Chiari Study (all CM1), 93 individuals from the PRSRC (all CM1 with syringomyelia), and 1,113 individuals from the HCP Young Adult Study (all controls). The SLCH cohort was retrospectively collected from a single clinical site and the RC Study prospectively collected an independent cohort from the research arm of the same institution. The ABCD Study enrolled from 21 research sites and data from the PRSRC was collected from 24 clinical sites. The HCP-YA Study enrolled from 2 research sites. In total, across all cohorts, this study analyzed data from 253 and 12,831 unique individuals in the CM1 and control groups, respectively, from 48 sites (Table 1).

**Table 1:** Demographics of all cohorts.

	Chiari	Controls
**SLCH clinical cohort**	34	34
Mean age (range)	10.5 (1.3–17.8)	10.2 (1.8–17.3)
Gender (M / F)	11 (32%) / 23 (68%)	17 (50%) / 17 (50%)
**ABCD study - baseline**	71	11,684
Mean age (range)	9.86 (8.98–10.97)	9.96 (8.32–11.28)
Gender (M / F)	36 (51%) / 35 (49%)	6,103 (52%) / 5,581 (48%)
**ABCD study - year 2**	47	7,997
Mean age (range)	11.99 (10.79–13.25)	12.07 (10.23–13.97)
Gender (M / F)	24 (51%) / 23 (49%)	4,313 (54%) / 3,684 (46%)
**ABCD study - year 4**	47	6,261
Mean age (range)	14.01 (12.79–15.52)	14.17 (12.51–16.58)
Gender (M / F)	26 (55%) / 21 (45%)	3,362 (54%) / 2,899 (46%)
**Redefining chiari study**	55	N/A
Mean age (range)	23.7 (4.5–53.3)	N/A
Gender (M / F)	18 (33%) / 37 (67%)	N/A
**PRSRC**	93	N/A
Mean age (range)	10.3 (1–21)	N/A
Gender (M / F)	36 (40%) / 53 (60%) **	N/A
**HCP-YA**	N/A	1,113
Age	N/A	22–35 *
Gender (M / F)	N/A	507 (46%) / 606 (54%)
**Total unique**	253	12,831

* Specific ages were not reported, only age ranges.

** Gender data unavailable for 4 subjects.

For the single site SLCH clinical cohort, the CM1 and control groups were matched by age with a mean of 10.5 years (range 1.3 – 17.8) and 10.2 years (range 1.8 – 17.3), respectively. At the time of this publication the ABCD Study is ongoing and scheduled to repeat MRI data collection every two years. At the time of this analysis complete data for the baseline timepoint and partial data for the year-2 and year-4 timepoints were available for analysis. Due to its study design the participants’ age is very uniform within approximately 2 years at each timepoint (Table 1). Data from the RC Study spanned a wider age range from pediatric to adults with a mean 23.7 years (range 4.5 – 53.3). PRSRC data spanned pediatric to young adult age ranges with a mean 10.3 years (range 1 – 21). The HCP-YA Study did not report specific ages per participant for privacy considerations and instead reported age ranges from 22–35 years for all participants.

Gender tended to have a female bias for studies enrolling CM1 participants, which is not uncommon for this population^39^. Proportion of females with CM1 in each study was comparable with 68% in the SLCH Cohort, 67% in the RC Study, and 60% in the PRSRC. The SLCH Control group was evenly balanced per the matched retrospective screening protocol. The ABCD and HCP-YA studies were also more evenly balanced due to prospective stratification in their study design.

**Fig. 2:**
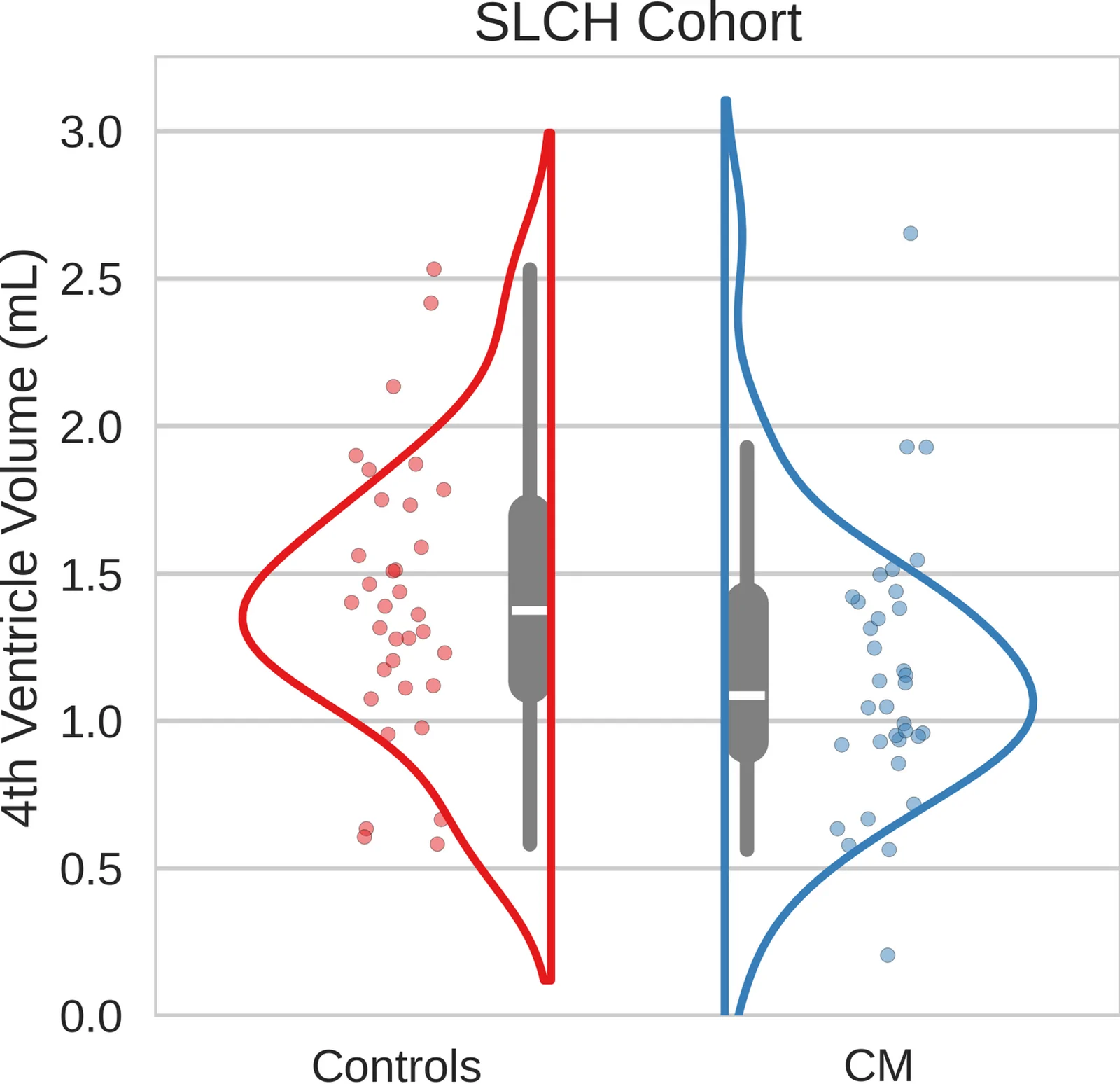
Smaller fourth ventricle volume measured by DL+DiReCT in individuals with CM1 compared to controls is demonstrated in this single site clinical dataset.

From the SLCH cohort we analyzed high resolution and high quality clinical MRI images processed by FreeSurfer and DL+DiReCT for automatic volumetric measurements (Fig. 2). The fourth ventricle volume corrected ratio was 0.82 (standard deviation 0.36) in individuals with CM1 (N=34), compared to a mean 1.0 (s.d. 0.37) in the control group (N=34). This difference represents a statistically significant smaller fourth ventricle associated with CM1 (t-statistic −2.025, p-value 0.047, Cohen’s d = 0.49). Uncorrected fourth ventricle volume showed a similar finding (Fig. 2) with a mean 1.15 mL (standard deviation 0.47) in individuals with CM1 compared to a mean 1.40 mL (s.d. 0.46) in the control group (t-statistic −2.249, p-value 0.028, Cohen’s d = 0.55).

**Fig. 3:**
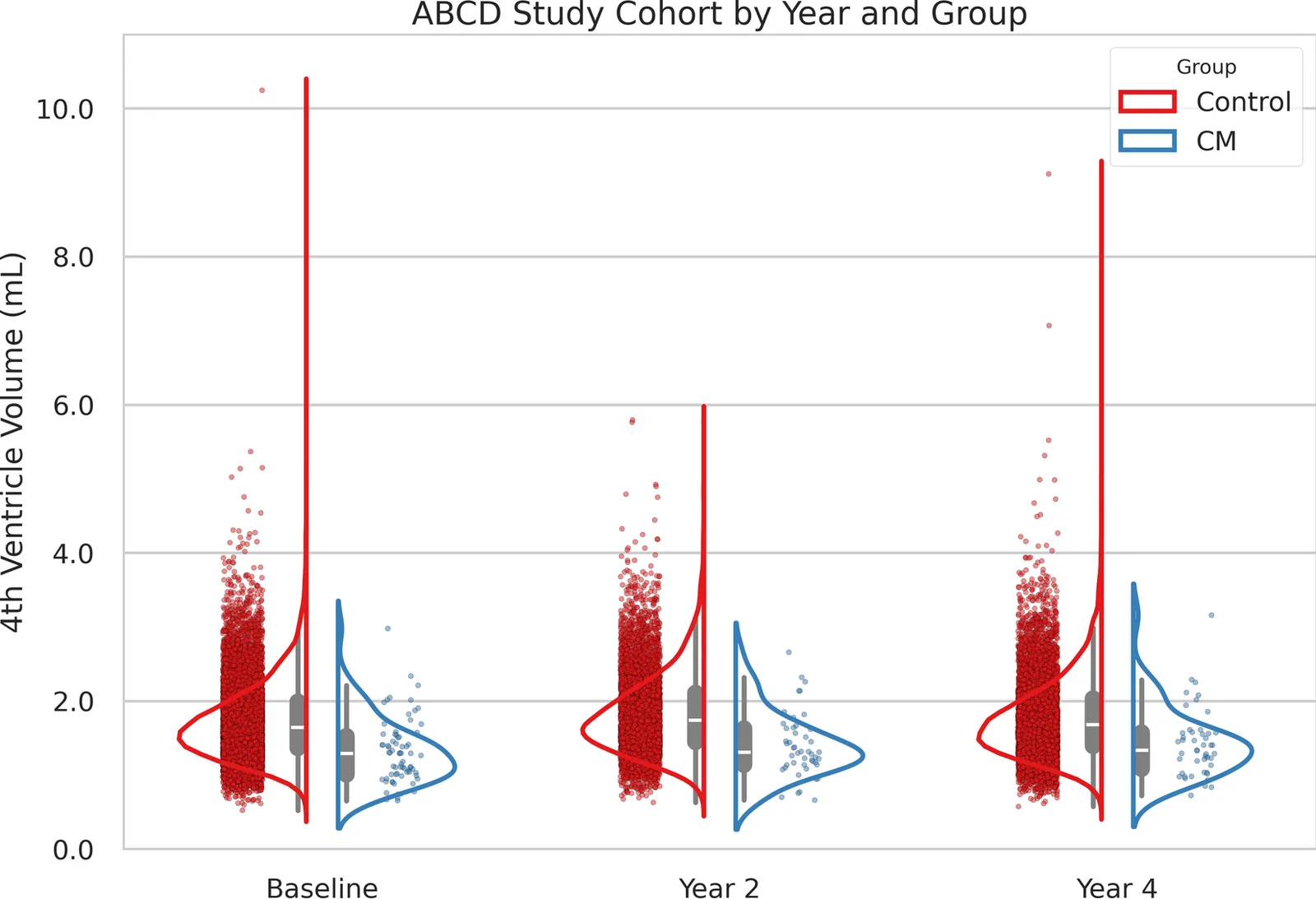
Fourth ventricle volume as segmented by FreeSurfer at baseline, Year 2, and Year 4 of the ABCD Study. Smaller fourth ventricle volume in individuals with CM1 compared to controls is a consistent finding at each timepoint.

In the ABCD Study cohort we first evaluated the fourth ventricle size as segmented by FreeSurfer and reported in the National Data Archive (NDA) release version 6.0. There are 71 subjects with CM1 as previously identified in the incidental findings study reported by Li and colleagues^26^. After excluding these 71 CM1 individuals from the total cohort, the remaining 11,684 individuals’ data were used as controls for the baseline timepoint. At 2-year and 4-year follow-up, respectively, data were available for analysis from 47 and 47 of the CM1 individuals and from 7,997 and 6,261 controls. At the baseline timepoint the fourth ventricle volume corrected ratio was 0.89 (s.d. 0.33) in the CM1 cohort compared to 1.16 (s.d. 0.33) in the control group (t-statistic = −6.901, p-value < 0.001, Cohen’s d = 0.82). At the year-2 longitudinal timpeoint the fourth ventricle volume corrected ratio was 0.95 (s.d. 0.33) in the CM1 cohort compared to 1.16 (s.d. 0.33) in the congrol group (t-statistic = −4.237, p-value < 0.001, Cohen’s d = 0.62). At the year-4 longitudinal timpeoint the fourth ventricle volume corrected ratio was 0.90 (s.d. 0.26) in the CM1 cohort compared to 1.20 (s.d. 0.34) in the congrol group (t-statistic = −5.99, p-value < 0.001, Cohen’s d = 0.88). Uncorrected fourth ventricle volumes showed similar findings at all three time points (Fig. 3) with a mean fourth ventricle volume 1.31 mL (s.d. 0.44) in the CM1 cohort compared to 1.72 mL (s.d. 0.51) in the control group at baseline (t-statistic = −6.832, p-value < 0.001, Cohen’s d = 0.81), 1.42 mL (s.d. 0.44) compared to 1.76 mL (s.d. 0.53) at year 2 (t-statistic = −4.402, p-value < 0.001, Cohen’s d = 0.64), and 1.39 mL (s.d. 0.43) compared to 1.82 mL (s.d. 0.53) at year 4 (t-statistic = −5.526, p-value = < 0.001, Cohen’s d = 0.81). Thus, the trend of smaller fourth ventricle volume in CM1 relative to Controls within the same timepoint persisted across all timepoints where ages are consistent within a narrow range.

**Fig. 4:**
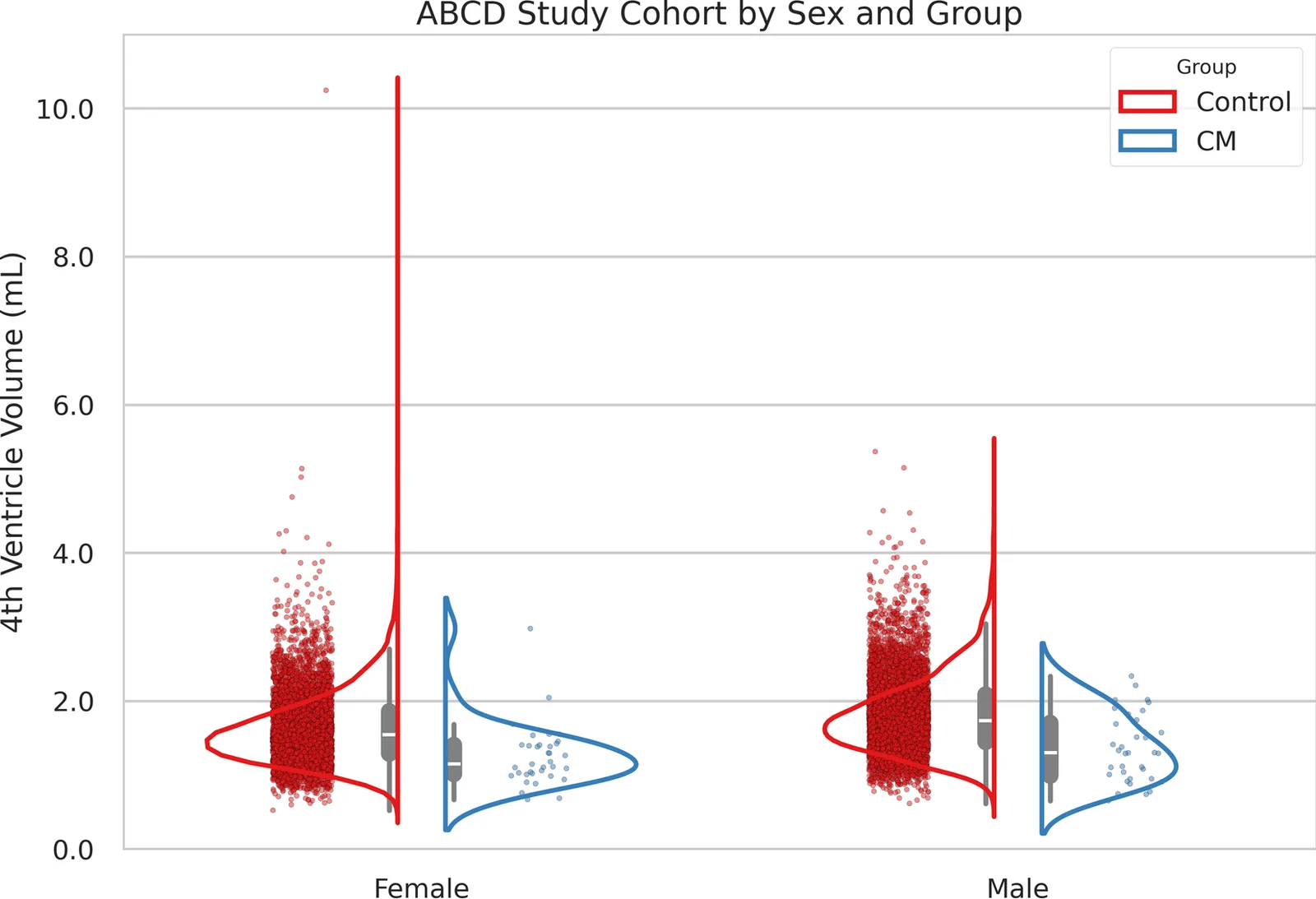
After separating participants in the ABCD Study by sex the fourth ventricle volume in CM is smaller for both females and males.

To test that this effect was seen in both female and male sex we repeated the analysis with the ABCD Study cohort at the baseline timepoint after separating by sex (Fig. 4). We saw a similar relationship of smaller fourth ventricle volume for both females (t-statistic = −4.538, p-value < 0.001) and males (t-statistic = −5.223, p-value < 0.001) with an effect size that was only slightly greater for males (Cohen’s d = 0.87) than females (Cohen’s d = 0.77).

Furthermore, given prior evidence to suggest multiple factors that may represent confounds in CM1^29^ we created one-to-one matched cohorts among the ABCD Study at the baseline timepoint. We matched on age, sex, ethnicity and race, and body-mass index (BMI). The matching procedure produced 57 matched subjects in each group. For the matched cohorts the mean fourth ventricle volume corrected ratio was 0.90 (s.d. 0.36) in the CM1 cohort compared to 1.19 (0.33) in the control group (t-statistic = −3.45, p-value = < 0.001, Cohen’s d = 0.65). Similar results are found from the uncorrected fourth ventricle volume which was 1.33 mL (s.d. 0.47) in the CM1 cohort compared to 1.69 mL (s.d. 0.49) in the control group (t-statistic = −4.019, p-value = < 0.001, Cohen’s d = 0.75).

**Fig. 5:**
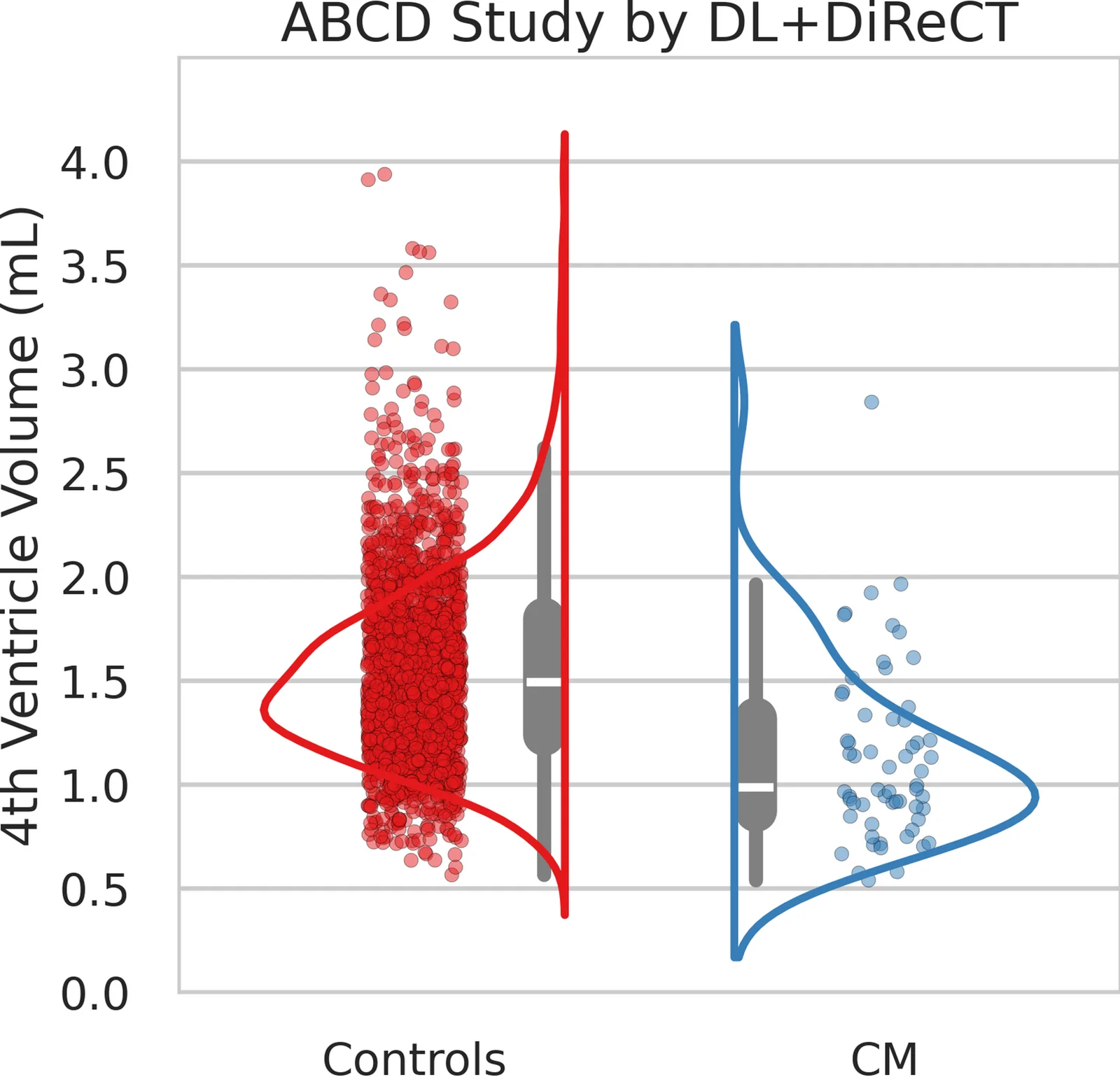
The fourth ventricle volume analysis was reproduced using an independent deep-learning tool for segmentation. The result held with this alternative segmentation technique demonstrating smaller fourth ventricle volume in individuals with CM1 compared to controls.

To reproduce our findings, we acquired and processed the T1-weighted MRI volumes from the baseline timepoint of the ABCD Study CM1 cohort, of which 60 from the original 71 were available, and a subset of the control subjects using an alternative software method for automated image segmentation. The subset we used for controls has been previously reported as a discovery cohort by selecting 1,964 subjects with known good quality imaging^30^. We processed these data using the DL+DiReCT pipeline to produce similar volumetric measurements from an independent software pipeline (Fig. 5). Using this alternative approach, we observed a fourth ventricle volume in the CM1 cohort (N=60) with a mean 1.13 mL (s.d. 0.42) compared to controls (N=1,964) with a mean 1.55 mL (s.d. 0.44), which reproduced the same result of a smaller fourth ventricle associated with CM1 (t-statistic −7.266, p-value < 0.001, Cohen’s d = 0.95). Thus, the DL+DiReCT method reproduced our findings with comparable effect sizes as FreeSurfer based segmentation.

**Fig. 6:**
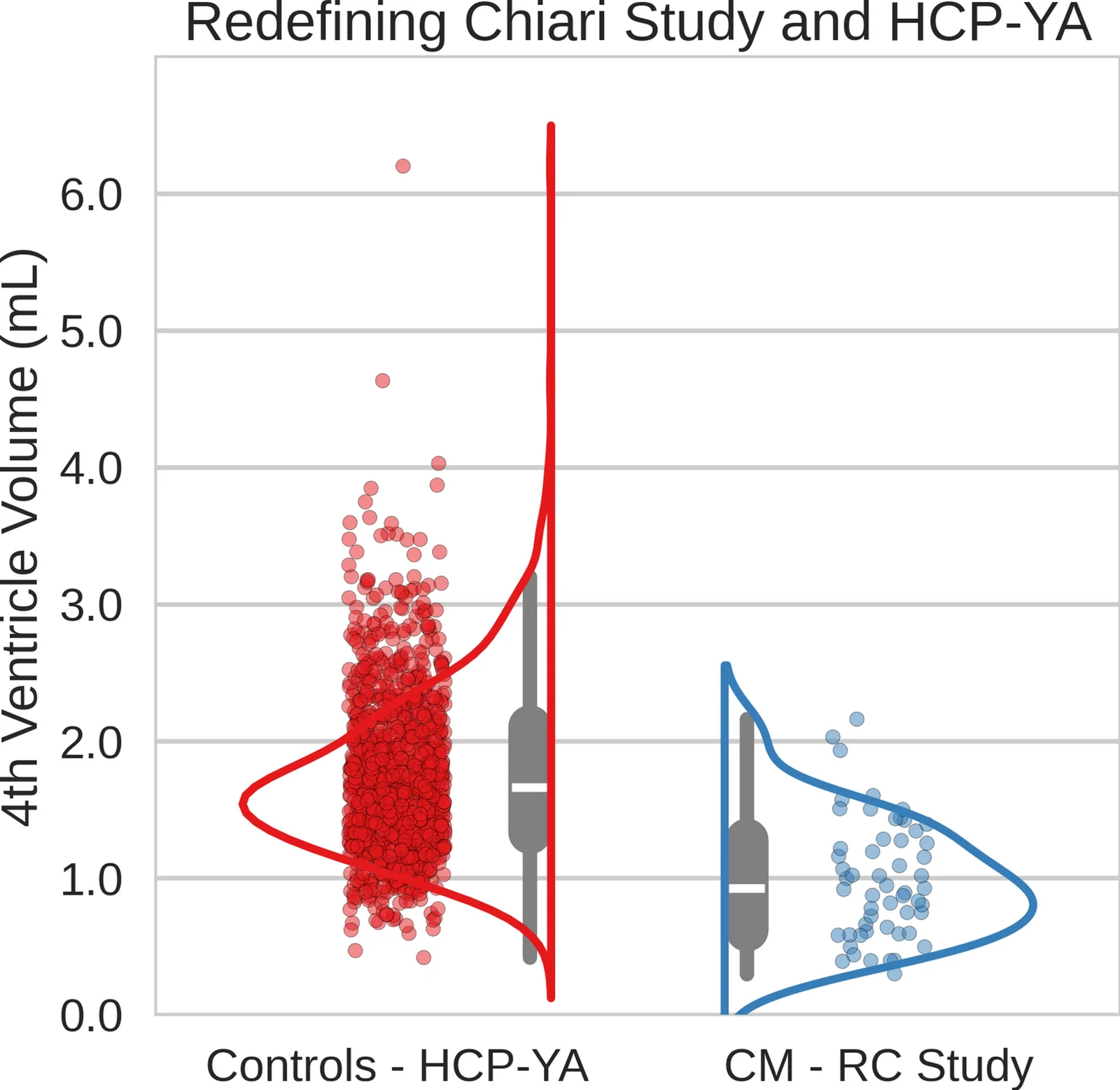
Replication by comparing prospective research MRI data from individuals with CM1 in the RC Study to control subjects in the HCP-YA Study. Smaller fourth ventricle is again replicated in this dataset.

The ABCD Study by design enrolled participants at a very narrow age range and selected for typically developing adolescents. Therefore, those with CM1 in this dataset represent incidental findings that may be subclinical. In contrast, the Redefining Chiari Study prospectively enrolled adolescents and adults with clinically diagnosed CM. Therefore, we sought to replicate our findings with the RC Study data (Fig. 6). As this study only enrolled clinically diagnosed individuals with CM1 and therefore did not include controls, we used imaging from the HCP-YA study for controls due to its comparable age range and similar MRI protocol. The fourth ventricle volume corrected ratio in CM1 subjects (N=55) from the RC Study (mean 0.69, s.d. 0.26) compared to controls (N=1,113) from the HCP-YA study (mean 1.12, s.d. 0.36) again replicated the consistent finding of a smaller fourth ventricle associated with CM1 (t-statistic −8.692, p-value < 0.001, Cohen’s d = 1.20). Uncorrected fourth ventricle volume measured by DL+DiReCT showed a similar finding (Fig. 6) with a mean 0.99 mL (s.d. 0.43 mL) for individuals with CM1 compared to a mean 1.76 mL (s.d. 0.60) for controls (t-statistic -9.333, p-value < 0.001, Cohen’s d = 1.29).

**Fig. 7:**
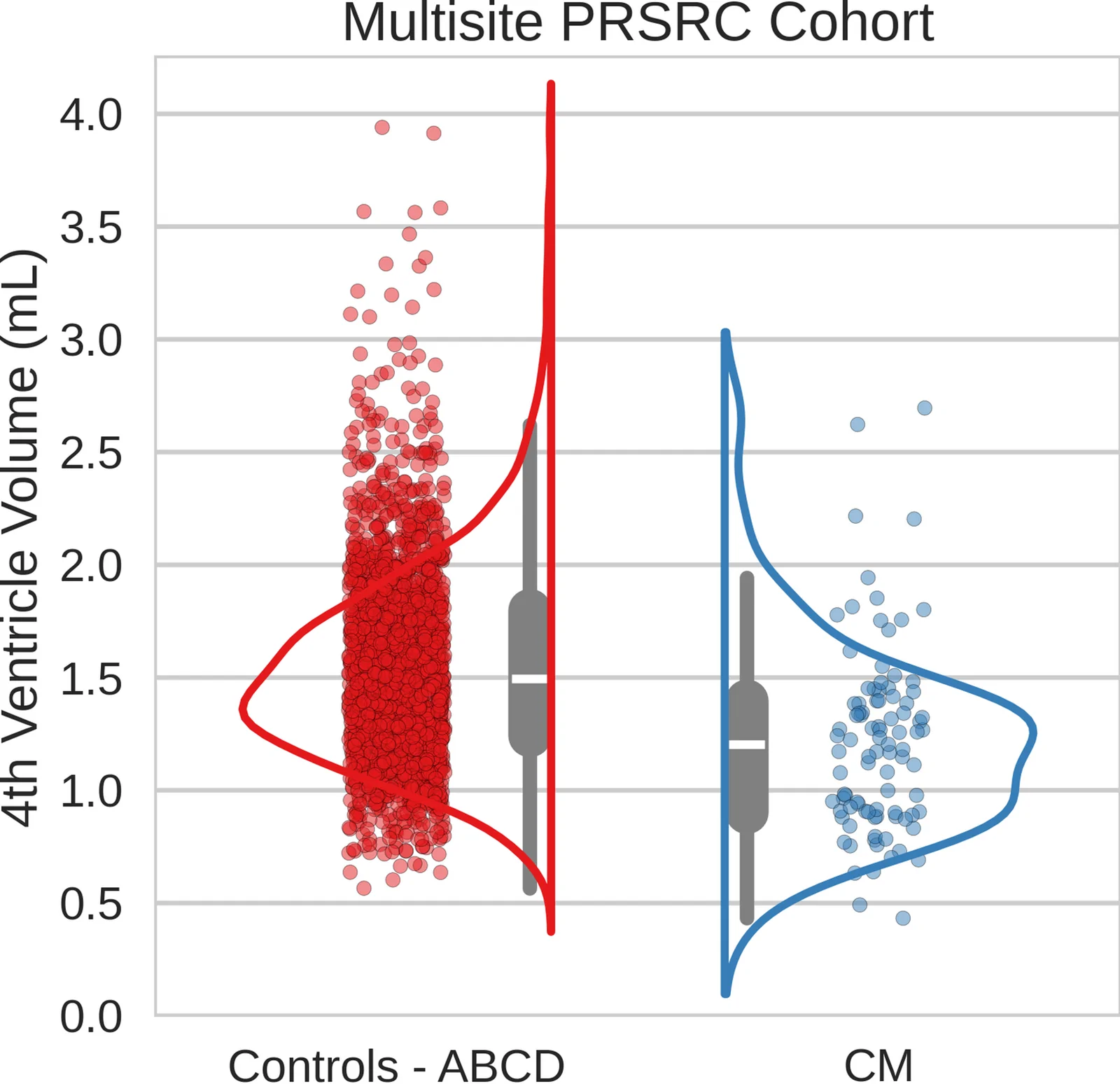
Replication in a multi-site clinical dataset acquired from the PRSRC. We again replicate finding smaller fourth ventricle volume in individuals with CM1 and syringomyelia compared to ABCD Study control group.

Next we sought to replicate this finding from a maximally heterogenous dataset that included clinical MRI scans from the 21 sites participating in the Park-Reeves Chiari and Syringomyelia Research Consortium (PRSRC). These data were collected retrospectively without attempt to standardize imaging acquisition but instead relied on real world imaging performed per each clinical site’s standard MRI protocol. After applying similar screening criteria as to our novel single site SLCH cohort (i.e., high quality, 1mm isotropic resolution, free of other abnormalities), we processed imaging from 93 individuals with CM1 and syringomyelia using the DL+DiReCT pipeline for volumetric analysis. As the PRSRC database does not include control data, we used the discovery control cohort from the ABCD Study baseline timepoint as reported above for comparison. In this multi-site PRSRC cohort we again replicated the consistent result of a smaller fourth ventricle volume corrected ratio in in CM1 (mean 0.86, s.d. 0.28) compared to controls (mean 1.16, s.d. 1.33), which were the full complement of controls from the ABCD Study baseline timepoint (t-statistic -8.573, p-value < 0.001, Cohen’s d = 0.89). Uncorrected fourth ventricle volume showed a similar finding (Fig. 7) of CM1 (mean 1.22 mL, s.d. 0.42) compared to controls (t-statistic −7.156, p-value < 0.001, Cohen’s d = 0.76).

Volume of some brain regions have been described to correlate with head size. Therefore, to test for a possible confounding effect of head size on 4^th^ ventricle volume we used the estimated total intracranial volume (eTIV) as calculated by FreeSurfer for the ABCD Study subjects. The effect sizes and inferential statistics did not meaningfully change at any timepoint after correcting for eTIV (baseline: d = 0.82, p-value < 0.001; year 2: d = 0.62, p-value < 0.001; year 4: d = 0.88, p-value < 0.001). The effect sizes and inferential tests for the original volumes and those corrective by eTIV are organized for comparison in (Table 2).

**Table 2:** Summary of results.

	CM1 group	Control group	Effect size	p-value
Freesurfer Corrected by eTIV	ABCD at Baseline	ABCD at Baseline	0.82	< 0.001
	ABCD at Year 2	ABCD at Year 2	0.62	< 0.001
	ABCD at Year 4	ABCD at Year 4	0.88	< 0.001
	PRSRC	ABCD at Baseline	0.89	< 0.001
	RC Study	HCP	1.20	< 0.001
	SLCH Clinical	SLCH Clinical	0.57	0.021
Freesurfer	ABCD at Baseline	ABCD at Baseline	0.81	< 0.001
	ABCD at Year 2	ABCD at Year 2	0.64	< 0.001
	ABCD at Year 4	ABCD at Year 4	0.82	< 0.001
	PRSRC	ABCD at Baseline	0.92	< 0.001
	RC Study	HCP	1.29	< 0.001
	SLCH Clinical	SLCH Clinical	0.49	0.047
DL+DiReCT	ABCD at Baseline	ABCD at Baseline	0.95	< 0.001
	PRSRC	ABCD at Baseline	0.76	< 0.001
	RC Study	HCP	1.29	< 0.001
	SLCH Clinical	SLCH Clinical	0.55	0.028

The unusually large size of the ABCD Study made for an excellent dataset to perform one-to-one matching between the smaller subset of CM1 subjects to a much larger pool of controls. However, such matching was not possible from the other datasets. Therrefore, we perormed a mutlivariable analysis including age and sex as covariates along with group (CM1 or control) on the fourth ventricle corrected ratio. For all datasets the Group consistently showed the largest effect. For the ABCD data at baseline the coefficients where largest for Group (0.267) with much smaller coefficients for sex (0.029) and age (0.002). For the PRSRC compared to ABCD discovery control cohort the coefficients where largest for Group (0.289) with much smaller coefficients for sex (0.029) and age (0.006). The HCP-YA data did not provide specific age, only a range, per subject therefore age was not included in the model when comparing the Redefining Chiari data to HCP-YA. For this model the coefficients where largest for Group (0.429) with much smaller coefficients for sex (0.029). While we are reassured by the consistent findings replicated across multiple independent datasets, this additional analysis provides further reassurance that the main effect is most attributable to CM1 as opposed to a age or sex as possible confounding variables.

## Discussion

In this study we present evidence supporting the association between CM1 and a smaller fourth ventricle. This association is replicated at the group level, but it is important to note that only a subset of CM1 subjects have a fourth ventricle volume below the mean of the respective control group. These results are strengthened by several methodological approaches strategically chosen to improve the robustness of our results. First, we capitalized on the availability of large amounts of imaging data available from multiple independent studies for a comprehensive analysis. The ABCD Study is the largest longitudinal imaging study of adolescents to date. At the time of this analysis the ABCD Study was ongoing but has enrolled nearly twelve thousand individuals who have committed to longitudinal imaging every two years. The tremendous number of individuals enrolled combined with prospective stratification across multiple sites makes the risk of sampling bias that could impact the sample distribution of fourth ventricle volumes unlikely to be impactful. Similarly, the longitudinal study design allows for confirmation that the finding is consistent within cohorts across timepoints. This within-group consistency across timepoints helps mitigate risk of measurement error skewing results. The highly consistent values over all three timepoints analyzed further support this conclusion, and the large corpus of data available at the time of this analysis makes it unlikely that future data releases will change the result.

The ABCD Study sought to enroll adolescents who were typically developing at baseline with the presumption that various neurologic conditions and psychopathologies would develop over the span of the longitudinal follow up in a subset of individuals. Given the breadth of enrollment several conditions would be expected to be captured by cross-sectional sampling. In this process 71 individuals were incidentally found to have a Chiari Malformation at baseline. More specifically, in the manuscript by Li and colleagues they defined CM1 as tonsillar ectopia that produced a triangular morphology of the cerebellar tonsils and separately identified other tonsillar ectopia. While these differences are insightful and have been described in other studies, our present analysis did not aim to differentiate morphology or other features of CM1 in this or any other dataset. Therefore, for this analysis both groups were combined as one CM1 cohort. Prior estimates of CM1 prevalence in the general population center around approximately 1% as diagnosed radiographically and 0.1% who are affected symptomatically^40^. Estimates generated from clinical imaging cohorts possibly overestimate the incidence due to selection bias of symptomatic individuals being more likely to seek medical care^41,42^. Because the ABCD Study enrollment criteria selected for typically developing adolescents, a selection bias towards asymptomatic individuals would be likely and may result in the observed prevalence of CM1 at 0.6% in this dataset to be less than that expected from the general population (i.e., the general population includes typically developing as well as those with neurologic conditions).

The initial description of tonsillar ectopia by Hans Chiari in 1891 was made by postmortem analysis of gross anatomy^1^. Subsequent advances in imaging technology transformed our ability to study human anatomy and with it came significant improvement in our understanding of normal and pathologic structure and function. One of the most impactful outcomes of imaging analysis on CM1 was the 5 mm rule that was codified in the pivotal study by Barkvoich and colleagues^43^. In their analysis the tonsil position was measured for 25 CM1 and 200 control individuals. Computer assisted technology was not available at that time and therefore measurements were performed by hand with mechanical calipers on hard copies of printed films. The images were acquired with a 0.5 or 0.6 Tesla MRI. In the almost 40 years that have passed since the Barkovich study, significant technological advances have transpired leading to our current study where imaging was obtained at 1 mm isotropic resolution with 3 Tesla MRI magnets followed by deep-learning volumetric segmentation performed on massively parallel high performance computing systems to analyze images from 13,084 individuals for robust generalizability. Despite the technological advances in the interim since the Barkovich study, the 5 mm rule has stood the test of time as it continues to dominate in clinical application for diagnosing and treating CM1. Beyond the singular measure of tonsillar ectopia, significant attention has more recently been focused on other facets of the posterior fossa and craniocervical junction. Studies associating CM1 and fourth ventricle volume are more recent but continue to be largely single center cohorts that are susceptible to replication failure.

The extant literature studying the association of fourth ventricle volume with CM1 has not been conclusive in establishing a well-defined relationship. Multiple single institution series analyzing retrospectively collected data have reported disparate findings. Seaman and colleagues studied 72 individuals with CM1 in comparison to 30 age-matched controls^17^. They analyzed volumes of the fourth and lateral ventricles as well as the posterior fossa. Their findings include larger fourth ventricle volume associated with CM1 compared to controls (1.31 vs 0.95 mL, p=0.012) and did not find a statistically significant difference in lateral ventricle of posterior fossa volumes. Alperin and colleagues studied multiple linear distances and volumetric measurements of MRI data from 36 CM1 and 37 control individuals^19^. Among other statistically significant findings they report smaller fourth ventricle in CM1 compared to controls (1.43 vs 2.19 mL, p<0.05). A study by Khalsa and colleagues similarly measured multiple linear distances and volumes from 51 symptomatic CM1 and 51 asymptomatic CM1 individuals from their institution and did not find a significant difference between any of their measures including fourth ventricle volume that was corrected by posterior fossa volume^20^. The largest single study in the literature by Kucukyildiz and colleagues reported linear orthogonal dimensions of the fourth ventricle in 251 individuals with CM1 with comparison to 273 controls^18^. Although not volumetric, they report larger anterior-posterior (10.51 vs 9.14 mm) and cranial-caudal (18.71 vs 17.01 mm) linear dimensions of the fourth ventricle in CM1 compared to controls. Their measure of left-right dimensions did not reach a statistically significant difference between groups.

It is possible that advances in MRI technology have improved ability to detect such differences and that prior studies did not have such a luxury. All imaging in this study was acquired with at least 1 mm resolution isotropic voxels. A difference in volume of 0.5 mL equates to 500 voxels in such an MRI. While it is not clear from this study what volume represents a clinically meaningful difference, we can be reassured that the difference in volume is not below the measurement precision of modern MRI acquisition. Each of these studies retrospectively analyzed data from their respective single center cohort with a single measurement technique. To address replicability, our study capitalized on multiple independent datasets from both single sites as well as data curated across multiple institutions including both prospective standardized research imaging protocols as well as retrospective clinical imaging. To generate a more robust and generalizable finding we reproduced results longitudinally within the ABCD dataset at three timepoints. We then replicated our results using ABCD data with an alternative FOSS tool for comparable volumetric measurement. Then we showed consistent reproducibility of the results in multiple independent datasets from multiple sites. Data from the PRSRC was the most diverse as it was collected from 24 clinical sites with different make and models of MRI scanners, different T1-weighted MRI sequences, and a broad age range spanning the full gamut of pediatric up to early adulthood, all of whom were diagnosed with CM1 and syringomyelia. Our finding of an association with smaller fourth ventricle in the maximally diverse PRSRC data confirms a robust result and supports its generalizability. It is possible that syringomyelia may have an association with fourth ventricle size and could therefore confound results of the PRSRC dataset, of which all individuals have syringomyelia. Collectively the data presented here garnered from multiple independent studies demonstrates generalizability across sub-clinical incidental CM1 (ABCD Study), symptomatic CM1 (SLCH cohort and RC Study), and CM1 associated with syringomyelia (PRSRC). This approach ensures replicability and reproducibility and maximizes the generalizability of our results beyond single site data and presents a more robust description of the effect of CM1 on fourth ventricle volume.

Rigor in this context refers to the application of systematic and meticulous methods to conducting research that help promote its validity. Without sufficient rigor, published data carries risk of Type 1 errors (false positives) that mistakenly identify a relationship that is unable to be replicated, or Type 2 errors (false negatives) that inadvertently fail to identify a relationship that truly exists. Enhancing replicability and reproducibility, core components of methodological rigor, ensures that research findings are reliable and can be consistently achieved under varying conditions. This consistency is crucial for confirming the validity of results and for building a robust foundation for future studies. When research is replicable and reproducible, it fosters greater confidence within the scientific community and among practitioners, ultimately leading to more effective and trustworthy applications in clinical practice. Moreover, it helps to identify and eliminate bias, reduces the likelihood of errors, and promotes transparency, thereby improving the overall quality and credibility of scientific inquiry.

It may be plausible that a not yet defined or confirmed latent variable may better describe clinically important variance in CM1 presentation and response to treatment. For example, findings published by Taylor and colleagues^44^ proposed a measure of how crowded the posterior fossa appears in differentiating types of CM1. Hofkes and colleagues^45^ proposed differentiating symptomatic and asymptomatic CM1 based on MRI measures of cerebrospinal fluid flow velocities. A study by Gupta and colleagues^42^ present the results of a large machine learning analysis that combined unsupervised clustering with expert opinion that generated three novel categories of CM1. It will need to be determined by future efforts if these, or other, novel metrics may help to explain clinical variance that currently is not well understood. Utilization of FOSS tools and open data for our study allows the application of our methods to sustain or refute these findings with novel cohorts defined in the future. Therefore, our approach acknowledges the field’s continued evolution as we collectively refine our understanding of CM1 and its many facets beyond the canonical tonsil ectopia.

Arachnoid veils of the fourth ventricle are a finding that has been reported during posterior fossa decompression with duraplasty and may have pathophysiologic implications to our findings of an associated with fourth ventricle size. Paik and colleagues analyzed a retrospective cohort of patients and found a statistically significant association between presence of arachnoid veil and the size of the syrinx, spinal canal, and thecal sac^9^. The possible disruption of CSF flow through the fourth ventricle outlet is implicated in their findings. The data analyzed in our study does not include status of arachnoid veils but is worth considering for future analysis.

## Limitations

In this study we made several specific methodological choices with the aim to maximize replicability and reproducibility. However, several important limitations could not be overcome in this study. Chiari malformations present with a wide range of features despite attempts to define sub-types. We acknowledge that even within CM1 sub-types there is likely to be a range of heterogenous presentations that may reflect different underlying pathophysiologic mechanisms. In this analysis we did not attempt to differentiate type 1 from type 0 or type 1.5, which may or may not have unique implications^47,48^. For example, previously published data report an association of type 1.5 with fourth ventricle roof angle^49^ and therefore could conceivably confound fourth ventricle volume. While a scoping analysis of all reported craniocervical morphometrics is beyond the scope of this analysis, it is worth noting that not all possible confounds have been accounted for.

This study focused on replication of findings across multiple datasets by comparing groups of CM1 to Controls. As such, its results should be interpreted as group level findings. We acknowledge that not every individual with CM1 has a fourth ventricle volume smaller than all other individuals in the Control group. While these group level findings help identify trends, that do not provide a high degree of subject specificity beyond the group level.

We did not aim to provide a scoping or systemic review of the literature and therefore it is likely that some data from similarly analyzed morphometrics may be missed in our discussion. Houston and colleagues similarly used the HCP data set for controls in their analysis of a much larger set of CM1 related morphometrics ^50^. Nwotchouang and colleagues similarly used the ABCD Study dataset to identify a novel group of individuals with CM1^29^. Our results should be interpreted within the context of other reported data.

It is a strength of the study that we found a consistent result across multiple datasets; however, not all datasets included all the same information regarding scanner make, model, and sequence parameters. Therefore, we were unable to model the effect of different MRI make, models, software versions, T1-weighted sequence parameters, or site effects on the main results. We believe it to be unlikely that a systematic effect on fourth ventricle size between CM1 and Control groups could be referable to such a confounder, but it is a limitation of this study that we could not test for this directly.

Furthermore, while some datasets have the strength of well-matched age groups (e.g., ABCD Study and SLCH clinical cohort), not all datasets could be well matched by age and other possible confounders. We believe the similar effects sizes found across all groups help reassure that low chance of a cofounding effect, but inability to accurately match by all possible confounds is a weakness of this study design. Similarly, being to show consistent results on test-retest at the group level at multiple timepoints in the ABCD Study is an important finding, yet we caution that unaccounted for bias in dropout across timepoints could potentially systematically alter the results.

## Conclusion

This study represents the largest volumetric assessment of the fourth ventricle in CM1 to date across local single site data and multiple large multisite datasets with and without syringomyelia. Across all datasets and longitudinal timepoints we consistently find smaller fourth ventricle volume associated with CM1. We employed robust methods to maximize replicability and reproducibility by using FOSS and open science methods. Collectively this large data analysis clarifies discrepant results in the extant literature and resolves the association of smaller fourth ventricle volumes associated with sub-clinical, symptomatic, and syringomyelia associated CM1 cohorts.

A smaller fourth ventricle is most consistent with overcrowding in the posterior fossa. Therefore, our findings are most consistent the size mismatch hypothesis in the pathogenesis of CM1. We do not rule out the possibility of multiple independent etiologies that could manifest in each unique individual leading to development of tonsil ectopia. However, these group level data provide support for overcrowding as a rational unifying theory to explain the association of smaller fourth ventricle and tonsil ectopia.

## Data Availability

Data from the ABCD Study are availabe via the NIH Brain Development Cohorts (NBDC) Data Sharing Platform (https://nbdc.nida.nih.gov/). Data from the HCP-YA Study are available from the Human Connectome website (https://www.humanconnectome.org/). Other data used in this analysis can be accessed via the asscociated Open Science Framework project page (https://osf.io/cn5ej/?view_only=99fe51ca00b84c178ab59e1a6cc53475).

## References

[1] Chiari, H. Ueber veränderungen des kleinhirns infolge von hydrocephalie des grosshirns1. *DMW-Dtsch. Med. Wochenschr.* **17**, 1172–1175 (1891).

[2] Tubbs, R. S., Iskandar, B. J., Bartolucci, A. A. & Oakes, W. J. A critical analysis of the Chiari 1.5 malformation. *J. Neurosurg. Pediatr.* **101**, 179–183 (2004). 10.3171/ped.2004.101.2.0179 15835105

[3] Iskandar, B. J., Hedlund, G. L., Grabb, P. A. & Oakes, W. J. The resolution of syringohydromyelia without hindbrain herniation after posterior fossa decompression. *J. Neurosurg.* **89**, 212–216 (1998). 9688115 10.3171/jns.1998.89.2.0212

[4] Shuman, W. H. et al. Is there a morphometric cause of Chiari malformation type I? analysis of existing literature. *Neurosurg. Rev.* **45**, 263–273 (2022). 34254195 10.1007/s10143-021-01592-4

[5] McGirt, M. J., Nimjee, S. M., Floyd, J., Bulsara, K. R. & George, T. M. Correlation of cerebrospinal fluid flow dynamics and headache in Chiari I malformation. *Neurosurgery* **56**, 716–721 (2005). 15792510 10.1227/01.neu.0000156203.20659.14

[6] Ibrahimy, A. et al. Association between resistance to cerebrospinal fluid flow near the foramen magnum and cough-associated headache in adult Chiari malformation type I. *J. Biomech. Eng.* **143**, 051003 (2021). 33454731 10.1115/1.4049788 PMC8086178

[7] Mugge, L. et al. Headache and other symptoms in Chiari malformation type I are associated with cerebrospinal fluid flow improvement after decompression: a two-institutional study. *World Neurosurg.* **163**, e253–e262 (2022). 35364297 10.1016/j.wneu.2022.03.108

[8] Heiss, J. D. Cerebrospinal fluid hydrodynamics in Chiari I malformation and syringomyelia: modeling pathophysiology. *Neurosurg. Clin. N. Am.* **34**, 81–90 (2023). 36424067 10.1016/j.nec.2022.08.007 PMC9708110

[9] Paik, K. S., Caudill, C., Arynchyna-Smith, A., Rocque, B. G. & Rozzelle, C. J. Predicting the presence of 4th ventricular outlet obstruction in Chiari I malformation. *Childs Nerv. Syst.* **40**, 2865–2870 (2024). 38847880 10.1007/s00381-024-06482-w PMC11322262

[10] Nyland, H. & Krogness, K. G. Size of posterior fossa in Chiari type 1 malformation in adults. *Acta Neurochir. (Wien)* **40**, 233–242 (1978). 676804 10.1007/BF01774749

[11] Nishikawa, M., Sakamoto, H., Hakuba, A., Nakanishi, N. & Inoue, Y. Pathogenesis of Chiari malformation: a morphometric study of the posterior cranial fossa. *J. Neurosurg.* **86**, 40–47 (1997). 8988080 10.3171/jns.1997.86.1.0040

[12] Sgouros, S., Kountouri, M. & Natarajan, K. Posterior fossa volume in children with Chiari malformation type I. *J. Neurosurg. Pediatr.* **105**, 101–106 (2006). 10.3171/ped.2006.105.2.101 16922070

[13] Botelho, R. V., Heringer, L. C., Botelho, P. B., Lopes, R. A. & Waisberg, J. Posterior fossa dimensions of Chiari malformation patients compared with normal subjects: systematic review and meta-analysis. *World Neurosurg.* **138**, 521–529 (2020). 32156591 10.1016/j.wneu.2020.02.182

[14] Goel, A. et al. Chiari 1 formation redefined-clinical and radiographic observations in 388 surgically treated patients. *World Neurosurg.* **141**, e921–e934 (2020). 32562905 10.1016/j.wneu.2020.06.076

[15] Goel, A. Is atlantoaxial instability the cause of Chiari malformation? outcome analysis of 65 patients treated by atlantoaxial fixation. *J. Neurosurg. Spine* **22**, 116–127 (2015). 25415487 10.3171/2014.10.SPINE14176

[16] Labuda, R. et al. A new hypothesis for the pathophysiology of symptomatic adult Chiari malformation type I. *Med. Hypotheses* **158**, 110740 (2022). 34992329 10.1016/j.mehy.2021.110740 PMC8730378

[17] Seaman, S. C., Dawson, J. D., Magnotta, V., Menezes, A. H. & Dlouhy, B. J. Fourth ventricle enlargement in Chiari malformation type I. *World Neurosurg.* **133**, e259–e266 (2020). 31513955 10.1016/j.wneu.2019.08.230

[18] Kucukyildiz, H. C. & Ozum, U. Fourth ventricle dimensions increase in chiari malformation type 1. *Turk. Neurosurg.* 10.5137/1019-5149.JTN.35404-21.2 (2021). 34859835

[19] Alperin, N. et al. Magnetic resonance imaging measures of posterior cranial fossa morphology and cerebrospinal fluid physiology in Chiari malformation type I. *Neurosurgery* **75**, 515–522 (2014). 25328981 10.1227/NEU.0000000000000507 PMC4854794

[20] Khalsa, S. S. S. et al. Morphometric and volumetric comparison of 102 children with symptomatic and asymptomatic Chiari malformation Type I. *J. Neurosurg. Pediatr.* **21**, 65–71 (2018). 29125445 10.3171/2017.8.PEDS17345

[21] Talari, K. & Goyal, M. Retrospective studies – utility and caveats. *J. R. Coll. Physicians Edinb.* **50**, 398–402 (2020). 33469615 10.4997/JRCPE.2020.409

[22] Casey, B. J. et al. The adolescent brain cognitive development (ABCD) study: imaging acquisition across 21 sites. *Dev. Cogn. Neurosci.* **32**, 43–54 (2018). 29567376 10.1016/j.dcn.2018.03.001 PMC5999559

[23] Hagler, D. J. Jr. et al. Image processing and analysis methods for the adolescent brain cognitive development study. *Neuroimage* **202**, 116091 (2019). 31415884 10.1016/j.neuroimage.2019.116091 PMC6981278

[24] Glasser, M. F. et al. The minimal preprocessing pipelines for the human connectome project. *NeuroImage* **80**, 105–124 (2013). 23668970 10.1016/j.neuroimage.2013.04.127 PMC3720813

[25] Fischl, B. et al. Whole brain segmentation. *Neuron* **33**, 341–355 (2002). 11832223 10.1016/s0896-6273(02)00569-x

[26] Li, Y. et al. Rates of incidental findings in brain magnetic resonance imaging in children. *JAMA Neurol.* **78**, 578–587 (2021). 33749724 10.1001/jamaneurol.2021.0306 PMC7985817

[27] Lawrence, B. J. et al. Cerebellar tonsil ectopia measurement in type I Chiari malformation patients show poor inter-operator reliability. *Fluids Barriers CNS* **15**, 33 (2018). 30554565 10.1186/s12987-018-0118-1 PMC6296028

[28] Geerdink, N. et al. Essential features of Chiari II malformation in MR imaging: an interobserver reliability study—part 1. *Childs Nerv. Syst.* **28**, 977–985 (2012). 22547226 10.1007/s00381-012-1761-5 PMC3376258

[29] Nwotchouang, et al. Imaging and health metrics in incidental cerebellar tonsillar ectopia: findings from the adolescent brain cognitive development study (ABCD). *Neuroradiology* **1924**, 1913–1924 (2021). 10.1007/s00234-021-02759-y PMC12678021 34247260

[30] Marek, et al. Identifying reproducible individual differences in childhood functional brain networks: an ABCD study. *Dev. Cogn. Neurosci.* **100706**, 100706 (2019). 10.1016/j.dcn.2019.100706 PMC6927479 31614255

[31] Van Essen, D. C. et al. The WU-Minn human connectome project: an overview. *Neuroimage* **80**, 62–79 (2013). 23684880 10.1016/j.neuroimage.2013.05.041 PMC3724347

[32] Feczko, E. *et al.* Adolescent brain cognitive development (ABCD) community MRI collection and utilities. *BioRxiv* 2021.07. 09.451638 (2021).

[33] Buckner, et al. A unified approach for morphometric and functional data analysis in young, old, and demented adults using automated atlas-based head size normalization: reliability and validation against manual measurement of total intracranial volume. *Neuroimage* **738**, 724–738 (2004). 10.1016/j.neuroimage.2004.06.018 15488422

[34] Rebsamen, M., Rummel, C., Reyes, M., Wiest, R. & McKinley,. Direct cortical thickness estimation using deep learning‐based anatomy segmentation and cortex parcellation. *Hum. Brain Mapp.* **4814**, 4804–4814 (2020). 10.1002/hbm.25159 PMC7643371 32786059

[35] Virtanen, P. et al. SciPy 1.0: fundamental algorithms for scientific computing in python. *Nat. Methods* **17**, 261–272 (2020). 32015543 10.1038/s41592-019-0686-2 PMC7056644

[36] Harris, C. R. et al. Array programming with NumPy. *Nature* **585**, 357–362 (2020). 32939066 10.1038/s41586-020-2649-2 PMC7759461

[37] The pandas development team. pandas-dev/pandas: Pandas. 10.5281/ZENODO.3509134 (2025).

[38] The psymatch development team. miaohancheng/pysmatch: psymatch. (2025).

[39] Waskom, M. L. Seaborn: statistical data visualization. *J. Open Source Softw.* **6**, 3021 (2021).

[40] Öhlén, E. et al. Difference in clinical presentation and surgical outcomes in pediatric and adult patients with Chiari malformation type 1: a single center retrospective study. *Acta Neurochir. (Wien)* **167**, 1–11 (2025). 40272545 10.1007/s00701-025-06534-3 PMC12021967

[41] Sadler, B. et al. Prevalence and impact of underlying diagnosis and comorbidities on Chiari 1 malformation. *Pediatr. Neurol.* **106**, 32–37 (2020). 32113729 10.1016/j.pediatrneurol.2019.12.005 PMC7156318

[42] Strahle, J. et al. Chiari malformation Type I and syrinx in children undergoing magnetic resonance imaging. *J. Neurosurg. Pediatr.* **8**, 205–213 (2011). 21806364 10.3171/2011.5.PEDS1121

[43] Aitken, L. A. et al. Chiari type I malformation in a pediatric population. *Pediatr. Neurol.* **40**, 449–454 (2009). 19433279 10.1016/j.pediatrneurol.2009.01.003 PMC3176758

[44] Barkovich, A. J., Wippold, F. J., Sherman, J. L. & Citrin, C. M. Significance of cerebellar tonsillar position on MR. *Am. J. Neuroradiol.* **7**, 795–799 (1986). 3096099 PMC8331977

[45] Taylor, D. G., Mastorakos, P., Jane, J. A. & Oldfield, E. H. Two distinct populations of Chiari I malformation based on presence or absence of posterior fossa crowdedness on magnetic resonance imaging. *J. Neurosurg.* **126**, 1934–1940 (2016). 27588590 10.3171/2016.6.JNS152998

[46] Hofkes, S. K. et al. Differentiation between symptomatic Chiari I malformation and asymptomatic tonsilar ectopia by using cerebrospinal fluid flow imaging: initial estimate of imaging accuracy. *Radiology* **245**, 532–540 (2007). 17890352 10.1148/radiol.2452061096

[47] Gupta, V. P. *et al.* (2025) Using Artificial Intelligence to Identify Three Presenting Phenotypes of Chiari Type-1 Malformation and Syringomyelia. *Neurosurgery* 10.1227 10.1227/neu.0000000000003249 39902903

[48] Liu, W. et al. No significant difference between chiari malformation type 1.5 and type I. *Clin. Neurol. Neurosurg.* **157**, 34–39 (2017). 28384597 10.1016/j.clineuro.2017.03.024

[49] Bordes, S., Jenkins, S. & Tubbs, R. S. Defining, diagnosing, clarifying, and classifying the Chiari I malformations. *Childs Nerv. Syst.* **35**, 1785–1792 (2019). 31049667 10.1007/s00381-019-04172-6

[50] Badrani, J. H. et al. Validation of fourth ventricle roof angle as a measure of brainstem dysfunction in pediatric Chiari malformation type I. *J. Neurosurg. Pediatr.* **36**, 694–701 (2025). 41072043 10.3171/2025.5.PEDS24552

[51] Houston, J. R. et al. A morphometric assessment of type I Chiari malformation above the McRae line: a retrospective case-control study in 302 adult female subjects. *J. Neuroradiol.* **45**, 23–31 (2018). 28826656 10.1016/j.neurad.2017.06.006

